# Correlation between diffusion tensor indices and fascicular morphometric parameters of peripheral nerve

**DOI:** 10.3389/fphys.2023.1070227

**Published:** 2023-02-23

**Authors:** Luka Pušnik, Igor Serša, Nejc Umek, Erika Cvetko, Žiga Snoj

**Affiliations:** ^1^ Institute of Anatomy, Faculty of Medicine, University of Ljubljana, Ljubljana, Slovenia; ^2^ Jožef Stefan Institute, Ljubljana, Slovenia; ^3^ Department of Radiology, Faculty of Medicine, University of Ljubljana, Ljubljana, Slovenia; ^4^ Clinical Institute of Radiology, University Medical Centre Ljubljana, Ljubljana, Slovenia

**Keywords:** magnetic resonance microscopy, peripheral nerve anatomy, fractional anisotropy, mean diffusivity, eigenvectors, diffusion tensor imaging

## Abstract

**Introduction:** Diffusion tensor imaging (DTI) is a magnetic resonance imaging (MRI) technique that measures the anisotropy of water diffusion. Clinical magnetic resonance imaging scanners enable visualization of the structural integrity of larger axonal bundles in the central nervous system and smaller structures like peripheral nerves; however, their resolution for the depiction of nerve fascicular morphology is limited. Accordingly, high-field strength MRI and strong magnetic field gradients are needed to depict the fascicular pattern. The study aimed to quantify diffusion tensor indices with high-field strength MRI within different anatomical compartments of the median nerve and determine if they correlate with nerve structure at the fascicular level.

**Methods:** Three-dimensional pulsed gradient spin-echo (PGSE) imaging sequence in 19 different gradient directions and *b* value 1,150 s/mm^2^ was performed on a 9.4T wide-bore vertical superconducting magnet. Nine-millimeter-long segments of five median nerve samples were obtained from fresh cadavers and acquired in sixteen 0.625 mm thick slices. Each nerve sample had the fascicles, perineurium, and interfascicular epineurium segmented. The diffusion tensor was calculated from the region-average diffusion-weighted signals for all diffusion gradient directions. Subsequently, correlations between diffusion tensor indices of segmentations and nerve structure at the fascicular level (number of fascicles, fascicular ratio, and cross-sectional area of fascicles or nerve) were assessed. The acquired diffusion tensor imaging data was employed for display with trajectories and diffusion ellipsoids.

**Results:** The nerve fascicles proved to be the most anisotropic nerve compartment with fractional anisotropy 0.44 ± 0.05. In the interfascicular epineurium, the diffusion was more prominent in orthogonal directions with fractional anisotropy 0.13 ± 0.02. Diffusion tensor indices within the fascicles and perineurium differed significantly between the subjects (*p* < 0.0001); however, there were no differences within the interfascicular epineurium (*p* ≥ 0.37). There were no correlations between diffusion tensor indices and nerve structure at the fascicular level (*p* ≥ 0.29).

**Conclusion:** High-field strength MRI enabled the depiction of the anisotropic diffusion within the fascicles and perineurium. Diffusion tensor indices of the peripheral nerve did not correlate with nerve structure at the fascicular level. Future studies should investigate the relationship between diffusion tensor indices at the fascicular level and axon- and myelin-related parameters.

## 1 Introduction

Certain medical conditions and penetrating injuries might cause individual nerve fascicles to be selectively more affected; therefore, accurate recognition of fascicle topography has the uttermost clinical importance (Härtig et al., 2018). As the pattern of fascicular involvement aid in the diagnostic workup of peripheral neuropathies, there is a great emphasis on its recognition. Clinical methods such as nerve conduction studies and electromyography are invasive, unpleasant, and give limited information. Accordingly, exploring available non-invasive radiologic modalities to extract information regarding nerve fascicular anatomy is imperative (Bilgen et al., 2005; Delgado-Martínez et al., 2016; Möller et al., 2018).

The application of clinical magnetic resonance imaging (MRI) for the depiction of peripheral nerves is increasing as it enables the assessment of peripheral neuropathies, nerve injuries or entrapments, and even tumors of the peripheral nerves (Chhabra et al., 2013; Bäumer et al., 2014). However, in trauma-related peripheral neuropathy, clinical MRI has limited accuracy in detecting pathologies, except in cases of severe nerve stretch or where the entire cross-section is affected (Eppenberger et al., 2014). Furthermore, when evaluating neoplasms of peripheral nerve with clinical MRI, differentiation of malignant from benign lesions can sometimes be difficult to achieve, even when there are characteristic signs of the malignancy (Chhabra et al., 2013). To surmount this obstacle, different strategies have been proposed. For example, the employment of advanced hardware, higher magnetic field magnets, and stronger gradients enabled the depiction of smaller structures as nerve fascicles; however, such studies are generally limited to *ex vivo* (Bilgen et al., 2005; Huang et al., 2015). Recently, specific MRI sequences such as fat-suppressed 3D fast low-angle shots have been proposed to improve the delineation of the nerve fascicles in healthy volunteers (Wang et al., 2023). Additionally, advanced techniques such as diffusion tensor imaging (DTI) have been exploited to further expand the options for depicting peripheral nerve pathologies (Khalil et al., 2008).

DTI enables measuring the effect of membranes on the apparent diffusion of water molecules. The specific arrangement of peripheral nerves results in diffusivity being predominantly directed along the axis of the nerve than in a perpendicular direction, thus being anisotropic. It is known that intact membranes are the primary determinant for anisotropic diffusion, with myelination having a modulating effect (Beaulieu, 2002). DTI measurements have been further utilized for deriving diffusion tensor (DT) indices that quantify the anisotropy (Pridmore et al., 2021). In rodent models, DTI has shown promise in distinguishing healthy, transected, and regenerating nerves (Lehmann et al., 2010). Moreover, even in macroscopic absent nerve discontinuity, DTI has been proven to detect minor nerve injuries (Boyer et al., 2015). DT indices also tend to correlate with behavioral changes and axonal density of rodents during the regeneration phases; therefore, they might serve as a promising tool in the future for recognizing unsuccessful nerve repair that requires further surgical intervention (Lehmann et al., 2010; Morisaki et al., 2011; Manzanera Esteve et al., 2019). In more recent research, DTI has also yielded a convenient tool for determining the severity of nerve injury in rats with the ability to distinguish different degrees of partial nerve transections (Manzanera Esteve et al., 2021).

As DT indices of peripheral nerve reflect the structural integrity of the nerve, they have attracted substantial attention for clinical application (Morisaki et al., 2011; Rangavajla et al., 2014). In clinical settings, it has been shown that demyelinating disease reflects in DT indices with the reduction of fractional anisotropy (FA), providing additional data regarding axonal degeneration in patients with peripheral neuropathies (Takagi et al., 2009; Kakuda et al., 2011). These data could complement clinical examination, electrophysiological evaluation, and conventional MRI for early recognition of patients with neuropathies who are eligible for neuroprotective therapies (Mathys et al., 2013). It has also been suggested that DTI could be an additional tool for assessing nerve compression syndromes, notably carpal tunnel syndrome (Khalil et al., 2008; Stein et al., 2009). In addition, DTI allows tractographic reconstructions; therefore, it can provide information on nerve integrity, predict the optimal approach of tumor resection, and predilect possible compromises in nerve impairment (Bruno et al., 2019).

Intra- and extra-fascicular structures of peripheral nerve likely possess different diffusion properties. It is unclear how this might affect clinical MRI scans, which provide an averaged diffusion-weighted signal. Notably, *in vivo* studies generally require image post-processing, which can affect the calculation of DT indices (Hiltunen et al., 2005; Stein et al., 2009). Accordingly, basic research is needed to further understand nerve DT indices obtained on clinical MRI. Depiction of the diffusion process at the fascicular level within different nerve compartments (fascicles, perineurium, and interfascicular epineurium) could enhance the understanding of nerve DT indices. High-field MRI is required to further depict such small structures. The present study aimed to obtain knowledge on the diffusion characteristics of different nerve compartments on high-field MRI and determine their relationship with nerve fascicular morphometric parameters.

## 2 Materials and methods

### 2.1 Sample preparation

A segment of the median nerve was obtained from the proximal upper arm of five fresh cadavers, less than 24 h *postmortem*. The cadavers were donated for research and educational purposes to the Institute of Anatomy, Faculty of Medicine, University of Ljubljana, through a willed cadaver donation program. Each nerve was cut into a 9 mm long segment, had carefully removed the surrounding connective tissue, and inserted in a 10-mm-diameter glass tube. To prevent sample dehydration, the glass tube was filled with perfluorinated liquid, Galden SV90 from Solvay (Brussels, Belgium), which does not produce any detectable MRI signal (Awais et al., 2022). The study was approved by the National Medical Ethics Committee of the Republic of Slovenia (Permit No: 0120–239/2020/3).

#### 2.1.1 Nerve donors

The donors were four females and one male, with a mean age of 75 years (range 70–80). The interval from death to nerve sampling ranged from 5 to 23 h. There was limited data regarding premortem medical conditions, but all subjects had an atherosclerotic disease and/or arterial hypertension with varying degrees of severity. None of the subjects had known peripheral nerve disease.

### 2.2 Magnetic Resonance Microscopy image acquisition

Magnetic Resonance Microscopy (MRM) was performed on a 9.4T (400 MHz proton frequency) wide-bore vertical superconducting magnet (Jastec Superconductor Technology, Tokyo, Japan) connected to an NMR/MRI spectrometer (Tecmag, Houston TX, United States). Before the imaging, the tube with the sample was inserted in a Micro 2.5 gradient system with a 10 mm RF probe (Bruker, Ettlingen, Germany) of the magnet.

DTI of the nerves was performed using a three-dimensional (3D) pulsed gradient spin-echo (PGSE) imaging sequence with diffusion gradients in 19 different directions; however, all with the same *b* value of 1,150 s/mm^2^. The selected *b* value was chosen given the preliminary results, whereas we have tested various *b* values up to 1,800 s/mm^2^. The selected value provided optimal conditions for measuring the leading eigenvalue within the nerve fascicles. The theory also supports the selected *b* value for the two-point experiment with *b*_1_ = 0 and *b*_2_ = *b* > 0, where the optimal *b* value is equal to *b* = 1.1/*D* (Xing et al., 1997). Acquisition of an additional reference *T*_2_-weighted image with no diffusion weighting (*b* = 0) was needed for DTI calculation. The images were acquired with the following parameters: TE/TR = 36/880 ms; δ = 3 ms; ∆ = 27 ms; G0 = 0.26 T/m; field of view 9 × 4.5 × 10 mm^3^; matrix size, 256 × 128 × 16; and 4 signal averages. The image resolution along the in-plane directions was 35 μm. Scanning was performed at room temperature of 21°C with a total acquisition time of 1 day 16 h.

### 2.3 Image analysis and nerve morphometry

**FIGURE 1:**
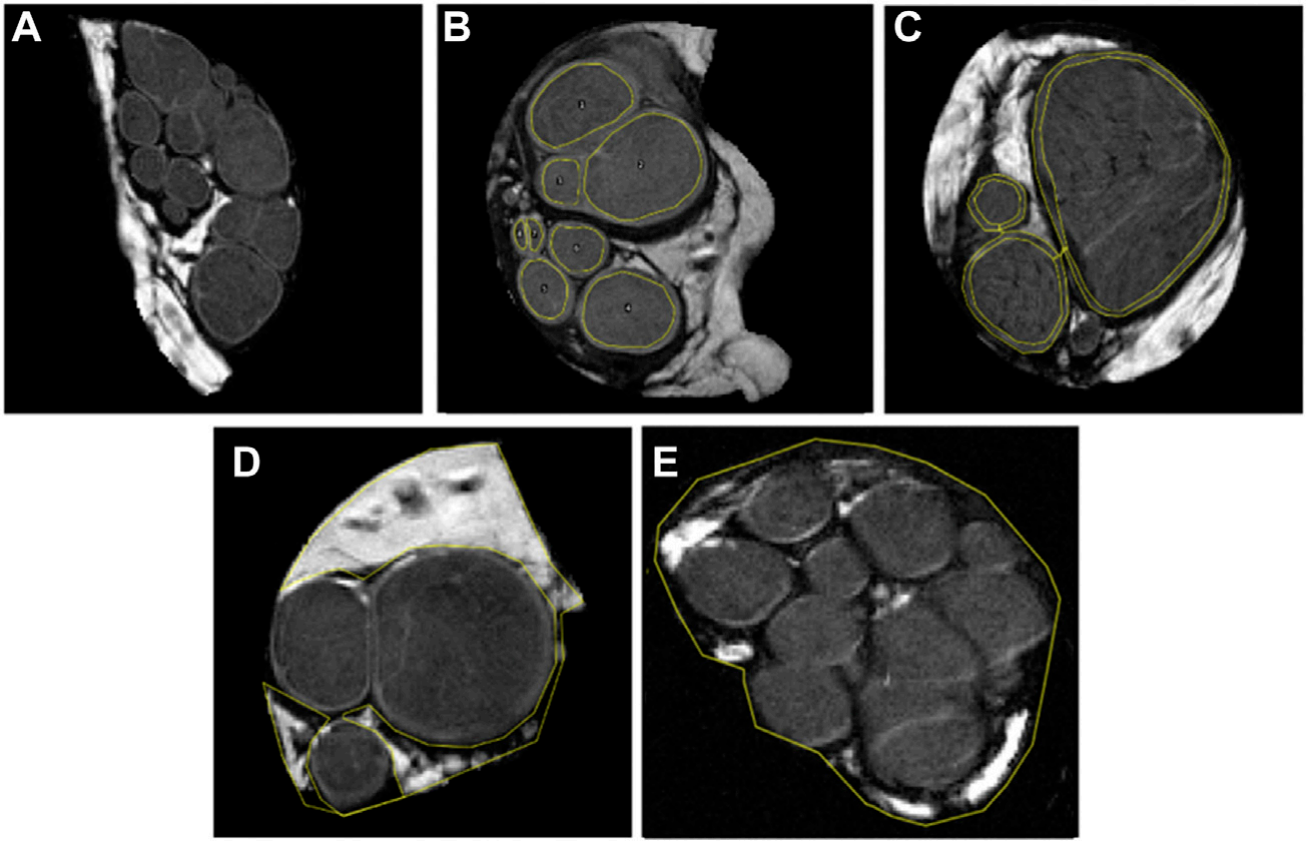
*T*_*2*_-weighed images, using *b*-value 0 s/mm^2^, displaying 0.625 mm thick representative slices of five analyzed median nerves. Note that each figure represents one out of sixteen slices. In figure **(A)** fascicles are sharply demarcated with a hyperintense line representing the perineurium. Further images depict the segmentation of **(B)** eight nerve fascicles, **(C)** thin layer of perineurium with two parallel lines, **(D)** interfascicular epineurium, and **(E)** nerve cross-sectional area.

The nerve segments of peripheral nerves, acquired in 16 continuous slices of 0.625 mm thickness, were identified on reference *T*_2_-weighted images (Figure 1A). Quality assessment of slices was performed, and slices with artifacts and partial volume effect were excluded from further analysis. In each included slice, fascicles, interfascicular epineurium, perineurium, and nerve cross-sectional area (CSA) were segmented. The fascicles were defined as intraneural hypointense oval- or round-shaped tissue circumferentially surrounded by a markedly hyperintense line representing the perineurium. The latter served as a reliable segmentation border (Figure 1B). The perineurium was segmented with a single measurement by two parallel lines, as shown in Figure 1C. The hyperintense tissue between the fascicles was defined as interfascicular epineurium (Figure 1D). The nerve was segmented to include the entire nerve but a minimal proportion of the background (Figure 1E). Segmentations were performed manually with the image processing software ImageJ (National Institutes of Health, Bethesda, Maryland, United States). The area was recorded for each segmentation and expressed as CSA for the nerve and fascicles. The fascicular ratio (FR) was calculated as a net fascicular CSA/nerve CSA, the ratio of perineurium as a net perineurium/nerve CSA, and the ratio of interfascicular epineurium as net interfascicular epineurium/nerve CSA (Tagliafico and Tagliafico, 2014).

The diffusion tensor was calculated from the acquired three-dimensional data as described previously (Basser et al., 1994; Awais et al., 2022). For each image voxel, the calculated diffusion tensor was diagonalized, which yielded maps of the tensor eigenvalues *D*_1_, *D*_2_, and *D*_3_ and of the corresponding eigenvectors ( ${\vec{\varepsilon }}_{1},{\vec{\varepsilon }}_{2}$ , ${\vec{\varepsilon }}_{3}$ ). Diffusion tensor and its diagonalization were also calculated for every delineated compartment from the corresponding average diffusion weighted signals of the compartment for 19 different diffusion gradient directions. Regional signal averaging enabled the calculation of the fractional anisotropy (FA), mean diffusivity (MD), and *D*_||_/*D*_⊥_ using the equations Eqs. 1–3 with less noise for each of the segmented compartments. The calculations were made using the software written in the C programming language, which has been previously developed and specifically modified by the authors (Awais et al., 2022).

$$MD=\frac{D_{1}+D_{2}+D_{3}}{3}$$ (1)

$$FA=\sqrt{\frac{3}{2}}\frac{\sqrt{{\left(D_{1}-MD\right)}^{2}+{\left(D_{2}-MD\right)}^{2}+{\left(D_{3}-MD\right)}^{2}}}{\sqrt{D_{1}^{2}+D_{2}^{2}+D_{3}^{2}}}$$ (2)

$$D_{\|}/D_{⊥}=\frac{D_{1}}{\frac{D_{2}+D_{3}}{2}}$$ (3)

### 2.4 Intra-observer agreement

A subset of 10 nerve fascicles, interfascicular epineurium areas, perineurial areas, and nerve CSA were randomly selected and segmented again by the same observer 30 days after the primary segmentation to assess intra-observer agreement. Intraclass correlation coefficient (ICC) was calculated from the FA (Koo and Li, 2016). FA was chosen for ICC calculation as this index is most commonly used DTI readout parameter in clinical environment reflecting the degree of cellular structure alignment (Kronlage et al., 2018).

### 2.5 Trajectory, diffusion ellipsoids, and color-coded ellipsoid/fiber orientation display

The DT data of the acquired nerve segments were displayed with trajectories and diffusion ellipsoids rendered with POV-Ray software (Persistence of Vision Pty. Ltd., version 3.7, Williamstown, Victoria, Australia) (Awais et al., 2022). The software generated a tractography display of the entire peripheral nerve length using the components of the first eigenvector. The subsequent slices were displayed with ellipsoids whose size and orientation correspond to the eigenvalues (size) and the eigenvectors (orientation).

### 2.6 Statistical analysis

Statistical analysis was performed using GraphPad Prism 9 (GraphPad Software Inc., San Diego, United States). The Shapiro-Wilk test was used to evaluate the groups for normality. Because normality and equal variance assumptions were met, the fascicular eigenvalues (*D*_1_, *D*_2_, and *D*_3_), as well as their derived indices (MD, FA, and *D*_||_/*D*_⊥_), were compared by two-way analysis of variance (ANOVA) followed by Tukey’s *posthoc* test when appropriate. When comparing DT indices of the perineurium, interfascicular epineurium, and nerve CSA one-way ANOVA followed by Tukey’s *posthoc* test was employed. The fascicular variability of FA was assessed using the coefficient of variation and then compared between fascicles and within fascicles with two-way ANOVA followed by Tukey’s *posthoc* test when appropriate. To determine the correlations between the DT indices and parameters at the fascicular level, linear regression was performed for each nerve sample then coefficients were compared using a one-sample *t*-test. The change of nerve FA in subsequent slices was calculated with linear regression. For the assessment of an intra-observer agreement, one-way ICC was used (Koo and Li, 2016). Differences were deemed statistically significant at *p* < 0.05. Data are given as means ± standard deviations, ranges, or percentages when appropriate.

## 3 Results

### 3.1 Nerve morphometric characteristics

After quality assessment, 56 image slices were included in the study (range of slices per nerve, 8–13). There were 7.94 ± 4.33 fascicles per slice with a mean fascicle CSA of 0.57 ± 0.66 mm^2^. The CSA of the nerve was 12.34 ± 3.53 mm^2^, and the FR was 0.46 ± 0.07. The ratio between perineurium/nerve CSA was 0.08 ± 0.03, and the ratio between interfascicular epineurium/nerve CSA was 0.46 ± 0.09.

### 3.2 DTI characteristics of nerve compartments

In the nerve fascicles, the eigenvalue *D*_1_, with an average of 0.81 ± 0.09·10^−9^ m^2^/s, was the highest and approximately 2-times higher than eigenvalues *D*_2_ or *D*_3_. The mean fascicular eigenvalue *D*_1_ was 27-times higher than in the interfascicular epineurium but 1.32-times lower than the mean eigenvalue *D*_1_ of the perineurium (Tables 1, 2, 3). There were significant differences between nerve samples regarding the fascicular and perineurium eigenvalues (*p* < 0.0001 and *p* < 0.0001, respectively), while there were no significant differences between nerve samples regarding the interfascicular epineurium eigenvalues.

**TABLE 1:** Diffusion tensor (DT) indices of nerve fascicles.

	*D*_1_ [·10^–9^ m^2^/s]	*D*_2_ [·10^–9^ m^2^/s]	*D*_3_ [·10^–9^ m^2^/s]	MD [·10^–9^ m^2^/s]	FA [0–1]	*D*_||_/*D*_⊥_ [0-∞]	N (n)
Nerve 1	0.77 ± 0.05	0.40 ± 0.05	0.37 ± 0.05	0.51 ± 0.04	0.41 ± 0.07	1.99 ± 0.28	12 (80)
Nerve 2	0.69 ± 0.10	0.35 ± 0.12	0.31 ± 0.12	0.45 ± 0.11	0.44 ± 0.13	2.22 ± 0.55	13 (40)
Nerve 3	0.90 ± 0.09	0.45 ± 0.07	0.41 ± 0.06	0.59 ± 0.04	0.43 ± 0.11	2.15 ± 0.29	8 (24)
Nerve 4	0.89 ± 0.13	0.50 ± 0.11	0.46 ± 0.10	0.62 ± 0.10	0.38 ± 0.08	1.89 ± 0.26	11 (109)
Nerve 5	0.78 ± 0.08	0.34 ± 0.04	0.29 ± 0.04	0.47 ± 0.03	0.52 ± 0.08	2.51 ± 0.28	12 (127)
x̄ + SD	0.81 ± 0.09	0.41 ± 0.07	0.37 ± 0.07	0.51 ± 0.09	0.44 ± 0.05	2.15 ± 0.24	Σ_N_ = 56
*p*-value^a^	<0.0001	<0.0001	<0.0001	<0.0001	<0.0001	<0.0001	Σ_n_ = 380

^a^ Comparison of means between different nerve specimens using two-way ANOVA; *D*_x_, eigenvalue; FA, fractional anisotropy; MD, mean diffusivity; N, number of slices analyzed; n, number of fascicles analyzed. Data are presented as mean (x̄) ± standard deviation (SD).

**TABLE 2:** Diffusion tensor (DT) indices of interfascicular epineurium.

	*D*_1_ [·10^–9^ m^2^/s]	*D*_2_ [·10^–9^ m^2^/s]	*D*_3_ [·10^–9^ m^2^/s]	MD [·10^–9^ m^2^/s]	FA [0–1]	*D*_||_/*D*_⊥_ [0-∞]	N
Nerve 1	0.03 ± 0.01	0.03 ± 0.01	0.02 ± 0.01	0.03 ± 0.01	0.13 ± 0.05	1.20 ± 0.07	12
Nerve 2	0.04 ± 0.02	0.03 ± 0.02	0.03 ± 0.02	0.03 ± 0.02	0.14 ± 0.05	1.20 ± 0.08	13
Nerve 3	0.04 ± 0.03	0.03 ± 0.02	0.03 ± 0.02	0.03 ± 0.02	0.14 ± 0.05	1.12 ± 0.11	8
Nerve 4	0.03 ± 0.02	0.03 ± 0.01	0.03 ± 0.01	0.03 ± 0.01	0.11 ± 0.04	1.12 ± 0.06	11
Nerve 5	0.03 ± 0.02	0.03 ± 0.01	0.03 ± 0.01	0.03 ± 0.01	0.12 ± 0.03	1.12 ± 0.06	12
x̄ + SD	0.03 ± 0.01	0.03 ± 0.00	0.03 ± 0.00	0.03 ± 0.00	0.13 ± 0.02	1.15 ± 0.04	Σ_N_ = 56
*p*-value^a^	0.79	0.91	0.91	0.88	0.37	0.38	

^a^ Comparison of means between different nerve specimens using one-way ANOVA; *D*_x_, eigenvalue; FA, fractional anisotropy; MD, mean diffusivity; N, number of slices analyzed. Data are presented as mean (x̄) ± standard deviation (SD).

**TABLE 3:** Diffusion tensor (DT) indices of perineurium.

	*D*_1_ [·10^–9^ m^2^/s]	*D*_2_ [·10^–9^ m^2^/s]	*D*_3_ [·10^–9^ m^2^/s]	MD [·10^–9^ m^2^/s]	FA [0–1]	*D*_||_/*D*_⊥_ [0-∞]	N
Nerve 1	1.06 ± 0.06	0.74 ± 0.01	0.55 ± 0.08	0.78 ± 0.04	0.33 ± 0.05	1.65 ± 0.10	12
Nerve 2	1.12 ± 0.12	0.92 ± 0.14	0.53 ± 0.10	0.83 ± 0.05	0.32 ± 0.10	1.54 ± 0.11	13
Nerve 3	1.10 ± 0.08	0.79 ± 0.12	0.54 ± 0.10	0.82 ± 0.05	0.34 ± 0.06	1.70 ± 0.20	8
Nerve 4	1.10 ± 0.05	0.73 ± 0.04	0.66 ± 0.06	0.84 ± 0.04	0.27 ± 0.03	1.54 ± 0.08	11
Nerve 5	0.96 ± 0.06	0.57 ± 0.07	0.41 ± 0.05	0.64 ± 0.04	0.40 ± 0.03	1.97 ± 0.17	12
x̄ + SD	1.07 ± 0.06	0.75 ± 0.13	0.53 ± 0.09	0.78 ± 0.08	0.33 ± 0.05	1.68 ± 0.18	Σ_N_ = 56
*p*-value^a^	<0.0001	<0.0001	<0.0001	<0.0001	0.0001	<0.0001	

^a^ Comparison of means between different nerve specimens using one-way ANOVA; *D*_x_, eigenvalue; FA, fractional anisotropy; MD, mean diffusivity; N, number of slices analyzed. Data are presented as mean (x̄) ± standard deviation (SD).

The mean MD followed the same pattern as eigenvalues, with the highest values calculated in the perineurial compartment. Compared to the fascicles, the perineurium had a 50.60% ± 20.72% higher MD, and the interfascicular epineurium had a 94.22% ± 0.80% lower MD (Tables 1, 2, 3). There were significant differences between nerve samples regarding the fascicular and perineural MD (*p* < 0.0001 and *p* < 0.0001, respectively), while there were no significant differences between nerve samples regarding the MD of the interfascicular epineurium.

The fascicle was the most anisotropic peripheral nerve compartment, with a mean FA of 0.44 ± 0.05. The coefficients of variation of fascicular FA throughout the same fascicle on sequential slices and between fascicles on the same slice were 0.17 ± 0.06 and 0.16 ± 0.03, respectively, and showed no statistically significant difference. Compared to the fascicles, the mean FA was lower in the perineurium (−23.95% ± 4.05%) and even lower in the interfascicular epineurium (−70.38% ± 3.91%). *D*_||_/*D*_⊥_ had the highest and most anisotropic values calculated in the fascicular compartment, while the interfascicular epineurium was the most isotropic compartment with a mean *D*_||_/*D*_⊥_ of 1.15 ± 0.04. Nerve samples differed significantly in FA and *D*_||_/*D*_⊥_ within the fascicles (*p* < 0.0001 and *p* < 0.0001, respectively) and perineurium (*p* = 0.0001 and *p* < 0.0001 respectively), while there were no significant differences in FA and *D*_||_/*D*_⊥_ of interfascicular epineurium (Tables 1, 2, 3).

DTI maps of five nerve segments providing eigenvalues (*D*_1_, *D*_2_, and *D*_3_), MD, and FA are included in the Supplementary Materials (Supplementary Figures 1–5).

### 3.3 DTI characteristics of nerve cross-section

The mean eigenvalues *D*_1_, *D*_2_, and *D*_3_ of the nerve were 0.46 ± 0.23·10^−9^ m^2^/s, 0.30 ± 0.15·10^−9^ m^2^/s, and 0.28 ± 0.14·10^−9^ m^2^/s, respectively, and differed significantly between the samples (*p* < 0.0001). Compared to the fascicular eigenvalues, mean nerve eigenvalues *D*_1_, *D*_2_, and *D*_3_ were 43.33% ± 23.07%, 27.87% ± 17.33%, and 24.45% ± 19.24% lower, respectively.

**FIGURE 2:**
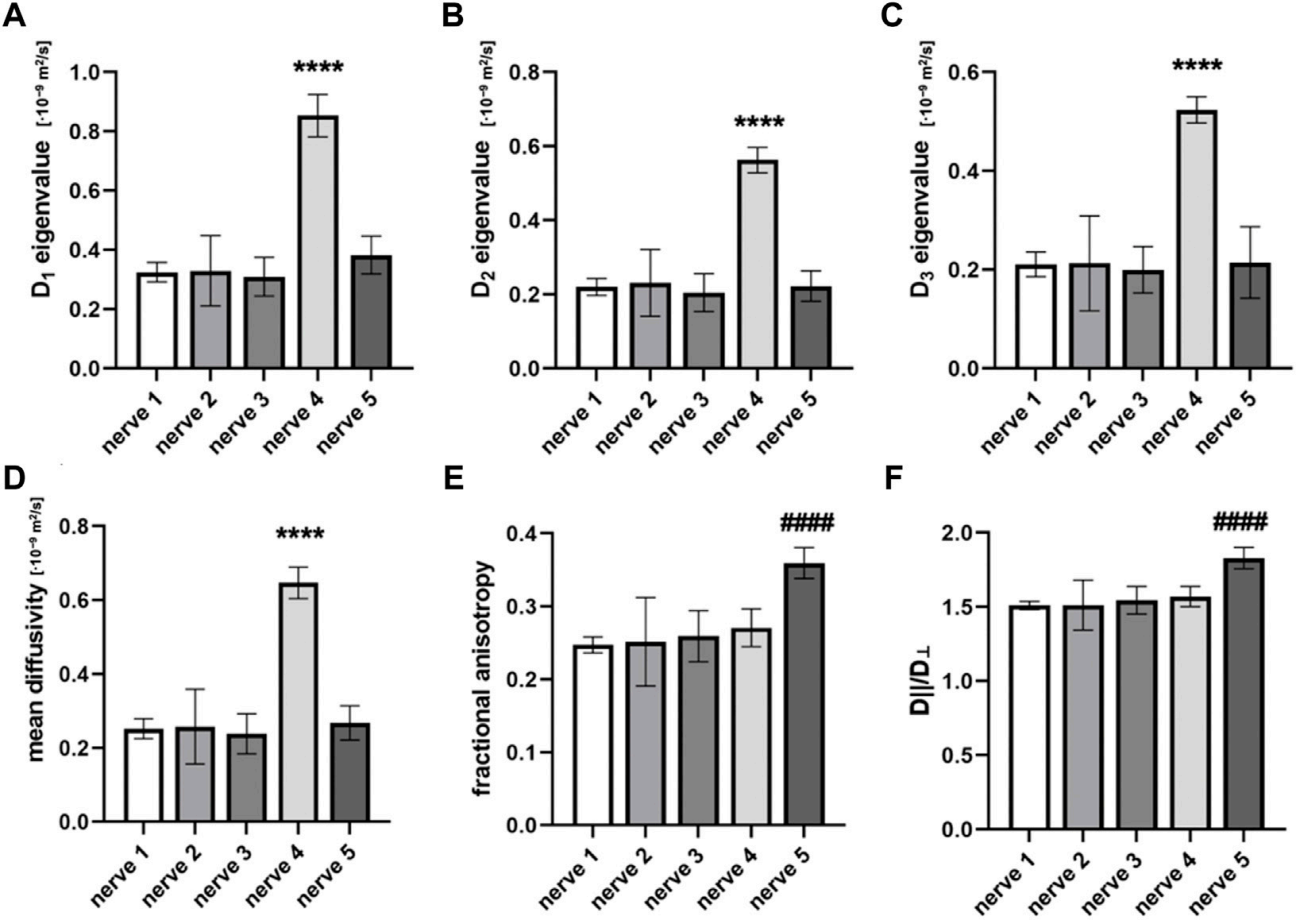
Diffusion tensor indices that are calculated from the cross-sectional area of different nerve samples. Figures compare **(A)** eigenvalue *D*_1_, **(B)** eigenvalue *D*_2_, **(C)** eigenvalue *D*_3_, **(D)** mean diffusivity (MD), **(E)** fractional anisotropy (FA), and **(F)** *D*_||_/*D*_⊥_. Data are presented as means and standard deviations. ****p < 0.0001 compared to nerves 1, 2, 3, and 5; ^####^ *p* < 0.0001 compared to nerves 1, 2, 3, and 4.

The MD of the nerve was 0.34 ± 0.17·10^−9^ m^2^/s. This was approximately 11-times higher than the MD of the interfascicular epineurium but 1.51 and 2.29-times lower than the nerve fascicles and perineurium, respectively. The MD differed significantly between the nerve samples (*p* < 0.0001) (Figure 2D).

**FIGURE 3:**
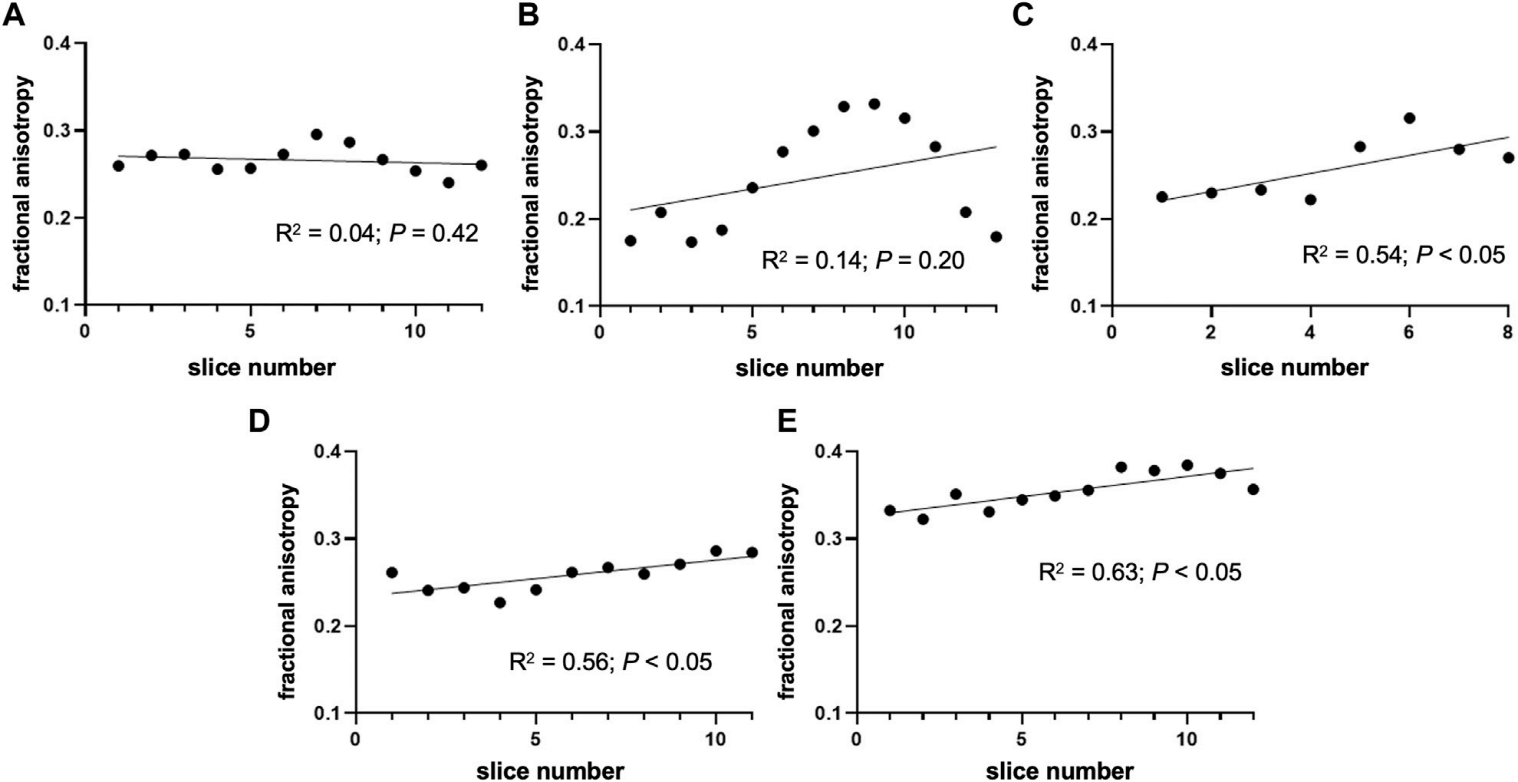
Change of fractional anisotropy throughout the nerve segments. The figure depicts the fractional anisotropy of the nerve cross-sectional area in consecutive slices. **(A)** Nerve sample 1 and **(B)** nerve sample 2 had no change of FA detected throughout the segment; however, statistically significant correlations were observed in **(C)** nerve 3, **(D)** nerve 4, and **(E)** nerve 5. Note that the orientation of the nerve (i.e., proximal/distal part) was not tracked during the sample preparation process.

The mean FA of the nerve was 0.28 ± 0.04. The highest FA was noted in nerve sample 5 (Figure 2E), which differed significantly from others (*p* < 0.0001). The mean coefficient of variation calculated from the FA of nerve samples was 0.12 ± 0.07. The nerve FA was approximately one-third and one-sixth lower than the FA of fascicles and perineurium, respectively, and 2-times higher than the FA of the interfascicular epineurium. The FA of the fascicles and perineurium measured together was 22.85% ± 7.75% higher than the nerve FA. Nerve samples 1 and 2 had no change of FA detected throughout the nerve segments; however, statistically significant correlations were observed in nerves 3–5 (Figure 3). *D*_||_/*D*_⊥_ showed the same pattern as FA with the highest values in nerve 5 (Figure 2F), which differed significantly from other samples (*p* < 0.0001).

### 3.4 Correlations between DT indices and nerve structure at the fascicular level

When evaluating the correlation between the nerve FA and the average FA of all fascicles on the same slice, we noted a moderately strong correlation (r = 0.74, *p* = 0.001). Additionally, we found a correlation between nerve FA and FA of the largest fascicle within all five nerves (r ≥ 0.47, *p* < 0.05).

However, we found no correlations when evaluating correlations between DT indices and structures at the fascicular level. No correlation was found between the DT indices of fascicles and CSA of fascicles (*p* ≥ 0.29). There was also no correlation between the mean FA of all fascicles on the slice and the number of fascicles (*p* = 0.59). We observed no correlation between the DT indices of nerve and the number of fascicles (*p* = 0.88). Additionally, there was no correlation between the nerve FA and FR (*p* = 0.34), and no correlation between the nerve FA and CSA of all fascicles on the same slice (*p* = 0.68).

### 3.5 Intra-observer agreement

ICC calculated from FA of the fascicles, and the nerve CSA showed excellent intra-observer agreement, 0.98 and 0.99, respectively. Good intra-observer agreement was observed for the perineurium, 0.89, and interfascicular epineurium, 0.86.

### 3.6 Anisotropic diffusion in nerves presented with tractography and diffusion ellipsoids

**FIGURE 4:**
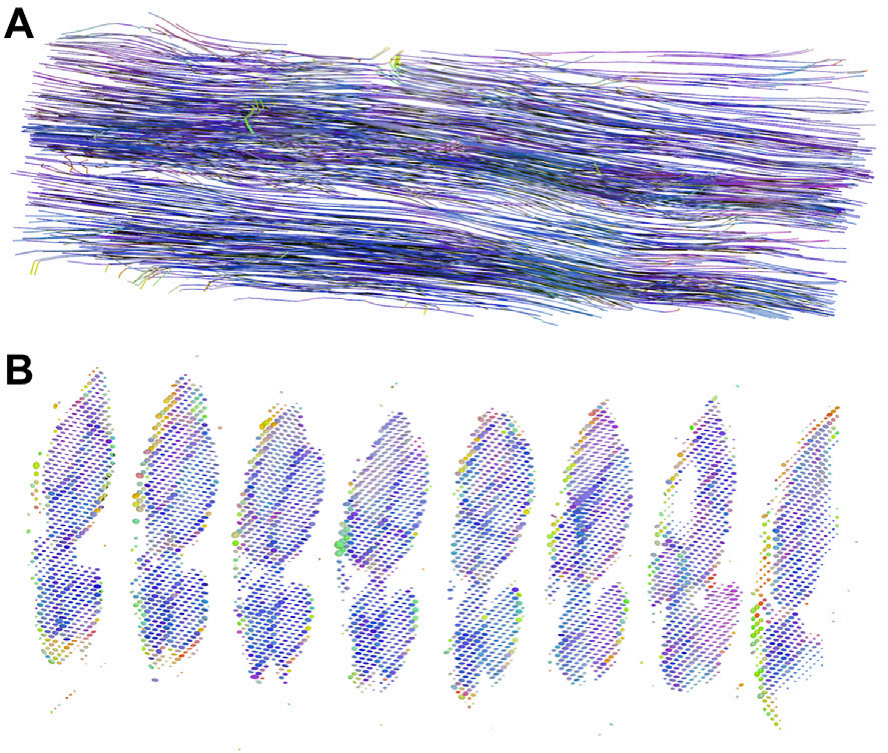
DT tractography and diffusion ellipsoids of one nerve sample. **(A)** The figure displays a three-dimensional representation of nerve fibers in an approximately 9 mm long segment of the median nerve. By convention, red color represents the *x*-direction, green color the *y*-direction, and blue color the *z*-direction; in our case, the nerve was oriented along the *z*-axis. **(B)** Diffusion ellipsoid display of the subsequent slices. The dimensions and orientations for the ellipsoids correspond to the eigenvalues and eigenvectors, while their color scheme is orientation-dependent and determined by the same principle as the colors in the tractography image.

DT data was demonstrated with graphic displays. Tractographic displays were generated using the fastest diffusion direction, which was oriented longitudinally along the course of the nerve. The three-dimensional representation of anisotropic nerve fibers of one nerve sample is shown in Figure 4A. As seen, nerve fascicles can be tracked along the entire nerve segment, their fiber density remains constant, and the fibers intermingle within the fascicles.

The presentation with diffusion ellipsoids shows ellipsoids that are oriented in the direction of the fastest diffusion (along the first eigenvector). The main axis of the ellipsoid provides information about the main diffusion direction in the voxel, while the shape of the ellipsoid gives the information about the degree of anisotropy. The difference in the eigenvalues within the fascicles and perineurium enables both compartments to be depicted and separated (Figure 4B). Note different eigenvectors of perineurium and fascicles that differ in values and orientations. In contrast, the interfascicular epineurium with considerably slower and isotropic diffusion cannot be adequately visualized. This anatomical compartment can be partially visualized in the background, where it can be seen as small dots of different colors and shapes.

## 4 Discussion

In our study, DTI in the magnetic field strength of 9.4T was employed on fresh *ex vivo* human median nerves, and DT indices of all major nerve anatomical structures were quantified. The anisotropic diffusion was shown throughout the long axis of the nerve, and nerve fascicles proved to be the most anisotropic nerve compartment. The DT indices of fascicles and perineurium differed significantly among subjects, while the interfascicular epineurium with the slowest and practically isotropic diffusion had no inter-subject differences. The DT indices of the peripheral nerve and anatomical compartments of the nerve did not correlate with nerve structure at the fascicular level.

DTI of peripheral nerves in the upper and lower extremities has been previously performed (Eppenberger et al., 2014; Kronlage et al., 2018; Godel et al., 2019). In the upper extremity, most investigations have been performed on the median nerve as it is the most frequently affected nerve in upper extremity entrapment neuropathies (Snoj et al., 2022). Several studies have depicted tractographic images of healthy median nerves and showed that Wallerian degeneration reduces the attenuation of trackable nerve fibers (Hiltunen et al., 2005; Khalil et al., 2008; Takagi et al., 2009; Boyer et al., 2015). More recent studies have predominately focused on the calculation of DT indices of nerve, mainly the FA (Kronlage et al., 2018; Awais et al., 2022). The nerve FA might vary along the longer nerve segment. Yao and Gai reported no change in FA along the length of the median nerve in the carpal tunnel (Yao and Gai, 2009), while the few other researchers demonstrated a decreasing trend of FA from proximal to distal locations near the carpal tunnel (Hiltunen et al., 2005; Guggenberger et al., 2013). Stein et al. (2009) showed that FA could differ significantly in a few centimeters long nerve segment. In our study, the FA did not diminish or augment throughout the nerve segment in two subjects, while the other three subjects had a trend of changing FA in the subsequent slices.

The mean FA of healthy median nerve reported in more extensive meta-analysis was 0.58 (Rojoa et al., 2021). In the wrist of healthy individuals, the mean FA was found to be in a broader interval between 0.48–0.71 (Kabakci et al., 2007; Stein et al., 2009; Barcelo et al., 2013; Guggenberger et al., 2013). Only the scarcity of studies have measured the FA of nerves in the upper arm and reported values in a similar interval range (Kronlage et al., 2018; Godel et al., 2019). It has been previously shown that the age of the subject is an important determinant for nerve FA (Tanitame et al., 2012; Kronlage et al., 2018). When accounting for this factor, the FA of the median nerve in our study was approximately two times lower compared to the FA of healthy peripheral nerves of similarly aged subjects in a study by Kronlage et al. (2018). Several factors should be accounted for when interpreting lower FA in our study. The most important factors are likely environmental. In our study, the MRM acquisition was performed at room temperature. This caused the diffusion process to be approximately 40% lower than the diffusion process at body temperature and may partially explain the differences in the values of DT indices compared to indices measured in previous *in vivo* studies (Holz et al., 2000; Lehmann et al., 2010). In a few *ex vivo* studies, unfrozen or fixated nerves were used (Boyer et al., 2015; Awais et al., 2022). Sample preparation can have an important impact on its integrity. It has been shown that tissue fixation in formaldehyde can significantly decrease the FA of a heart muscle (Lohr et al., 2020). Conversely, Haga et al. (2019) did not observe a change in FA between fixed and non-fixed marmoset brains; however, the use of formaldehyde did significantly decrease eigenvalues and MD of the fixed brain. As there is a lack of data on how formaldehyde might affect DT indices of peripheral nerve, we have used a fresh nerve to exclude the effects of the fixative procedure or possible rupture of the nerve cells during the freezing/thawing cycle.

The outstanding FA in nerve 5 can be partly attributed to the low body mass index of this donor; nevertheless, such inter-individual differences can still be found between healthy individuals (Kronlage et al., 2018). As reported by Godel et al. (2019), increased radial diffusivity reflects damage to myelin integrity, whereas changes in axonal diffusivity might be more specific for axonal degeneration. In nerve 4, eigenvalues *D*_1_, *D*_2_, and *D*_3_ were equally increased which probably supports the hypothesis of a major role of environmental factors.

Differences in MR hardware and imaging protocols can also lead to discrepancies between studies. Most existing studies were performed on conventional 3T MRI (Stein et al., 2009; Barcelo et al., 2013; Guggenberger et al., 2013; Kronlage et al., 2018); however, some researchers have also applied 7T whole-body MRI systems (Riegler et al., 2016; Yoon et al., 2018). Although a high-field-strength system was used in our study, it has been previously shown on the brain that field strength has little effect on the FA (Zhan et al., 2013). Importantly, studies use scanners from different manufacturers, and it has been shown that FA of the median nerve in healthy individuals significantly differs within the wrist between different MR scanners (Guggenberger et al., 2013). Post-processing within *in vivo* studies involves applying threshold values to distinguish nerve from muscle fibers or ligaments (Hiltunen et al., 2005). In these studies, FA is calculated from the fiber tractography images, which normally have a high threshold value (Hiltunen et al., 2005; Kabakci et al., 2007; Stein et al., 2009; Barcelo et al., 2013). Thus, the impact of isotropic nerve compartments, such as interfascicular epineurium, that decreases FA of nerve CSA is excluded from the calculation to a certain degree. In our study, no threshold was applied to obtain the most reliable data. Consequently, the FA of fascicles and perineurium combined was approximately a quarter higher than the FA of the nerve, which included interfascicular epineurium.

The estimated signal-to-noise ratio (SNR) in the fascicular region was approximately 14 (*b* = 0 s/mm^2^). In comparison to the study by Yoon et al. (Yoon et al., 2018), this SNR was comparable to their *in vivo* 7T MRI system. However, our in-plane resolution was considerably better (35 μm vs. 120 μm), and slices were also thinner (0.625 mm vs. 2 mm). The resolution advantage in our experiment is due to the more sensitive receiver coil, which had only 10 mm in diameter; thus, the filling factor (nerve to coil diameter ratio) was high, and the signal reception was, therefore, considerably better than in the *in vivo* experiment at 7T where a surface coil was used. Some advantage was also due to a somewhat stronger magnetic field (9.4T vs. 7T). This comparison demonstrates that *in vivo* MR imagining at 7T is promising but still needs several improvements, especially in signal detection, to match MR microscopy results at higher fields and dedicated hardware (special gradient and signal receive coils).

The long scanning window of approximately 40 h posed a challenge concerning tissue desiccation. Immersion in formaldehyde would be optimal for preventing the autolytic process; however, it contains hydrogen atoms that produce a signal on MRI. Hence, each nerve was placed instantly after the excision into the fluorinated carbon liquid, which substantially reduced problems with stability and autolysis. In our previous pilot study (Awais et al., 2022), minor nerve shrinkage of one pixel was noted during the scanning of the nerve sample in this liquid. This issue was adequately addressed with an innovative scanning strategy by reordering the scanning loops, whereas all twenty images were acquired simultaneously, not sequentially. Thus, the influence of the sample volume change was evenly distributed among all images and did not importantly affect the calculations of DT indices.

In our study, fascicles proved to have the highest FA in the analysis of the nerve compartments. This result was expected due to the specific arrangement of nerve fibers in the peripheral nerve (Sunderland, 1978). It is well known that the fascicular pattern can change in submillimeter sections (Sunderland, 1945; Sunderland, 1978). When evaluating the FA within the same fascicle, we found no statistical difference between the fascicular coefficients of variation in sequential slices and the fascicles of the same slice. We hypothesize that the fibers crossing between the fascicles contributed to high fascicular coefficients of variation within the same fascicle and explain why no difference was observed (Figley et al., 2022).

The perineurium had only moderately lower FA than the fascicles. The latter provides a diffusion barrier made of concentrically flat perineural cells, which contributes to poorer membrane permeability for water molecules and could lead to higher FA (Hill and Williams, 2002). As the perineurium is thinner than the resolution of our MRM system (Reina et al., 2015), it is essential to consider the partial volume effect. The segmentation of perineurium might partially include highly anisotropic fascicles and isotropic interfascicular epineurium. The interfascicular epineurium, as a collagenous compartment of the extracellular matrix, does not form any non-permeable barrier. Hence, it had the lowest FA mean approaching isotropic diffusion when considering the influence of the SNR (Peltonen et al., 2013; Awais et al., 2022). Fascicle FA has an important impact on nerve FA; however, measured fascicular parameters did not affect the nerve FA. Previous studies have shown that higher FA correlates with increased axonal density, axonal diameter, and myelin density (Takagi et al., 2009). Thus, demyelinating disorders could contribute to differences in fascicular FA between subjects included in this study (Kakuda et al., 2011). As seen, the FA was not correlating with structures at the fascicular level; therefore, axon- and myelin-related parameters tend to have a more important role in diffusion (Takagi et al., 2009).

We acknowledge that this study had some limitations. First, we had limited clinical data on the subjects from whom we obtained nerve samples. Consequently, the differences in DT indices between the subjects might have been even greater than they would have been between healthy subjects. It would be imperative in future research to expand the sample size and provide more clinical data about pathologies that could affect nerve integrity. Another limitation of this study is the acquisition at room temperature. As the environmental temperature could not be strictly controlled, minor fluctuations in room temperature could partially impact the DT indices of individual samples and cause important differences between the samples. A third limitation is that the determination of individual compartments could occasionally be ambiguous; hence, individual segmentations of the perineurium or smaller fascicles were more challenging to implement. This could lead to partially overlapping regions. Another limitation is a relatively long scanning time resulting in the subjection of nerve samples to the autolytic process. Accordingly, this has to be considered when compared to *in vivo* studies or *ex vivo* studies with shorter acquisition time. The last limitation is the limited ability to translate our results directly into the partial trauma series; non-etheless, we believe basic knowledge is essential for future understanding of how partial nerve transection reflects in a change of DT indices within different nerve compartments. Moreover, the understanding of diffusion within the nerve compartments can be translated into the nerve entrapment syndromes (where oedema is present) or diabetic neuropathy and thus help evaluate how individual compartments contribute to the anisotropy change.

## 5 Conclusion

High-resolution DTI depicted highly anisotropic diffusion within the fascicles and perineurium. The interfascicular epineurium had more isotropic diffusion. As median nerve DT indices did not correlate with nerve structure at the fascicular level, future studies should investigate their relationship with axon- and myelin-related parameters.

## Data Availability

The raw data supporting the conclusion of this article will be made available by the authors, without undue reservation.
