# Supplementary figures and images for: Correlation between diffusion tensor indices and fascicular morphometric parameters of peripheral nerve
Source: Front Physiol. 2023 Feb 23;14:1070227. doi: 10.3389/fphys.2023.1070227 (PMC9995878; doi:10.3389/fphys.2023.1070227)

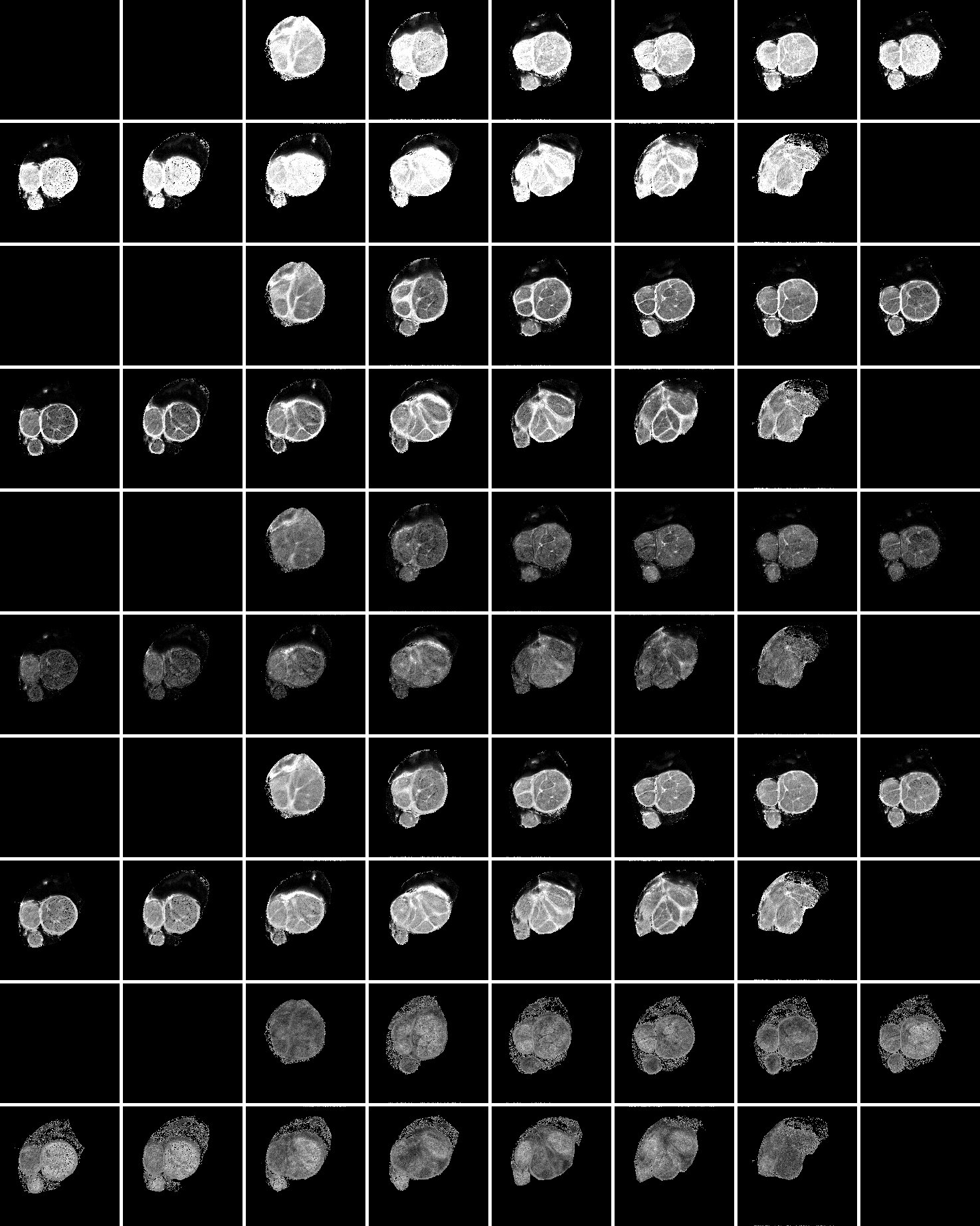

Supplement: Supplementary file 1 [file Image3.TIF]

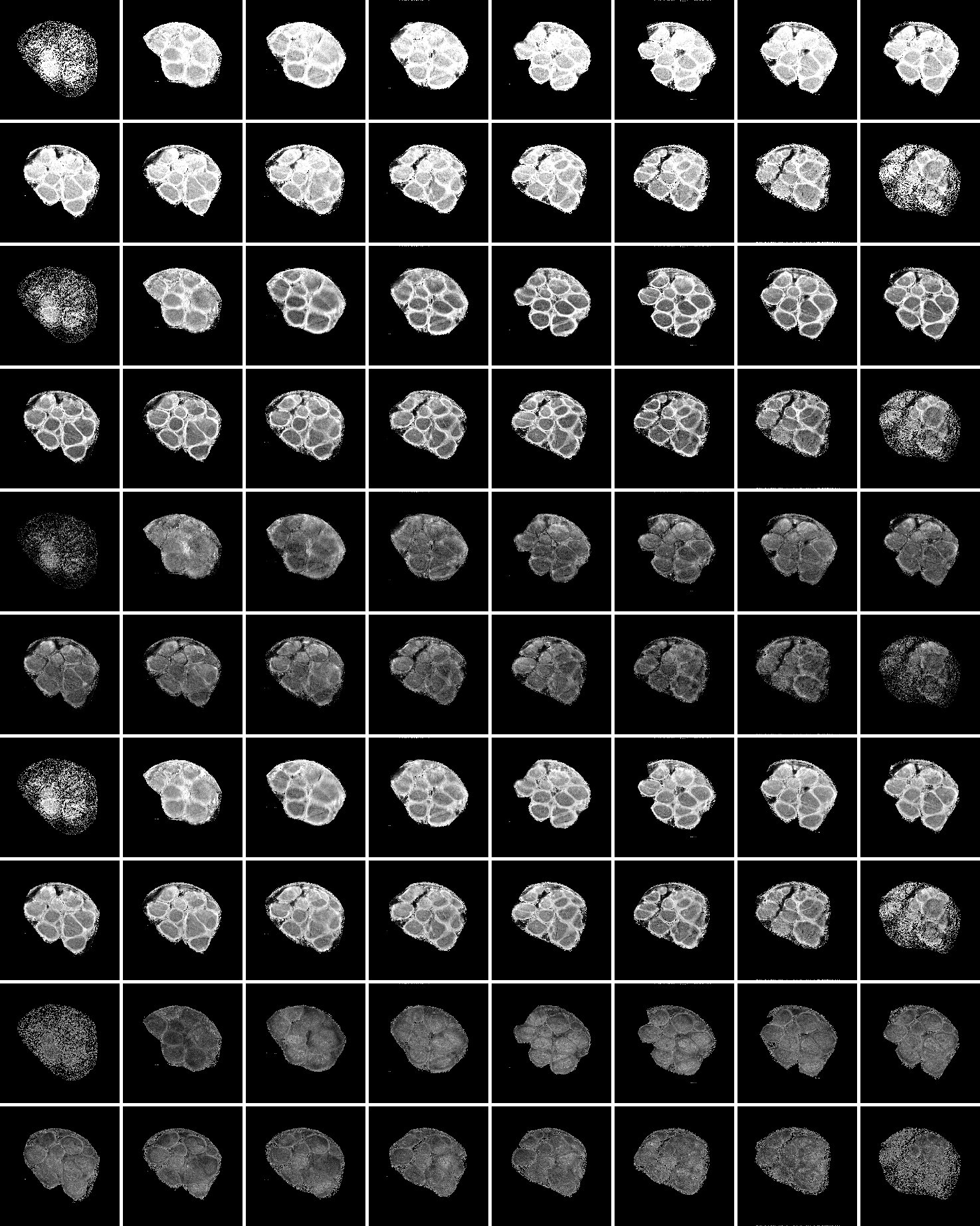

Supplement: Supplementary file 2 [file Image4.TIF]

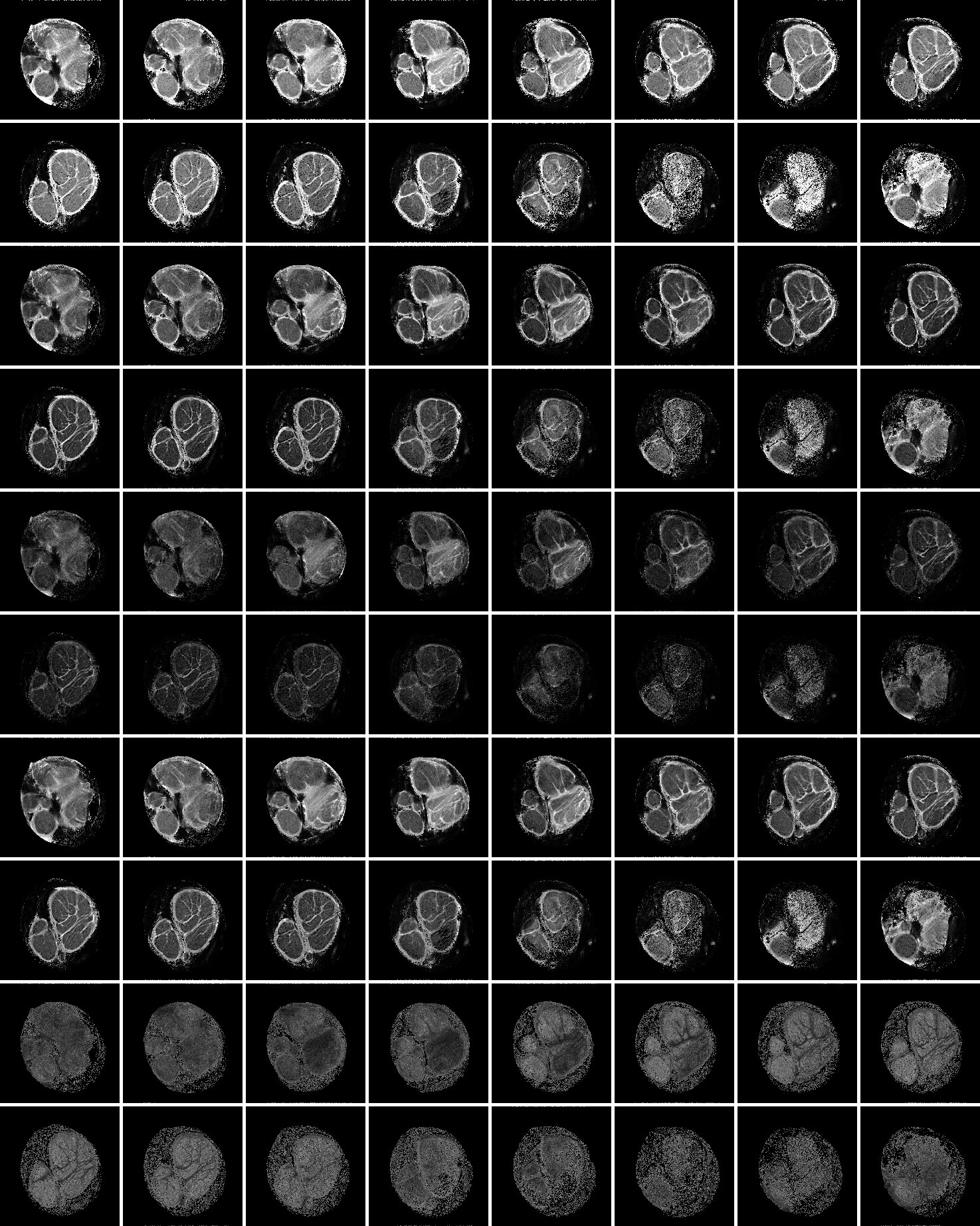

Supplement: Supplementary file 3 [file Image2.TIF]

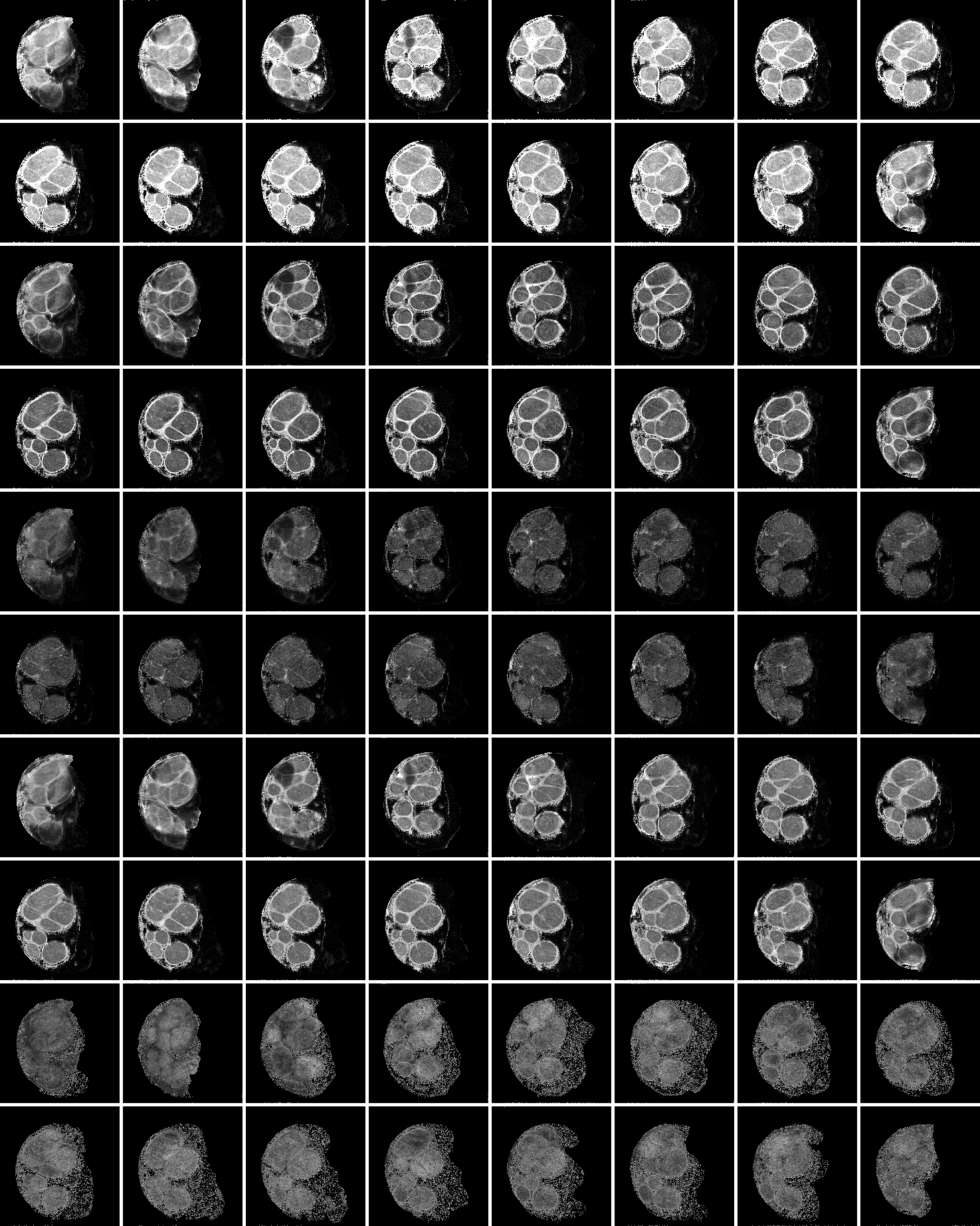

Supplement: Supplementary file 4 [file Image1.TIF]

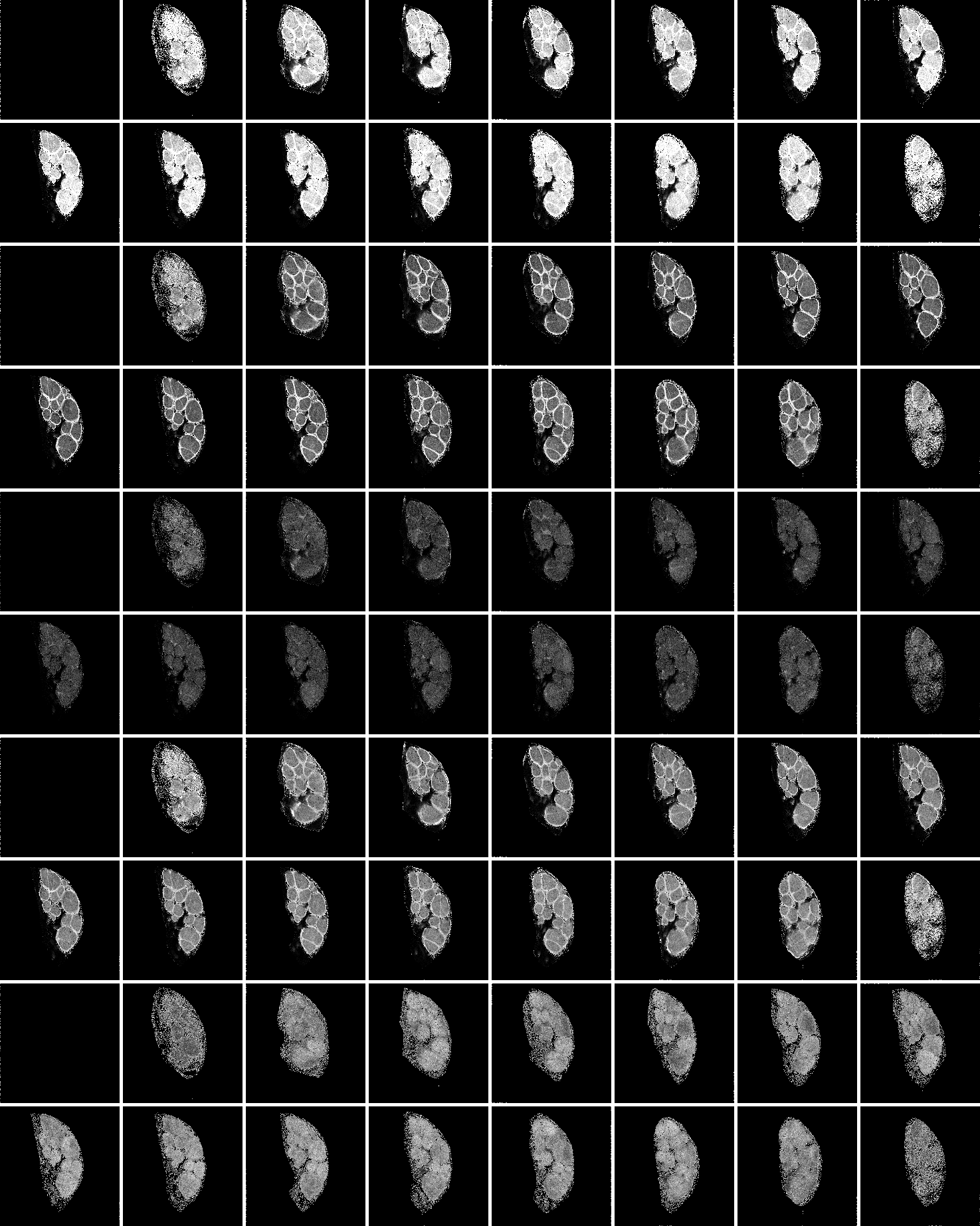

Supplement: Supplementary file 5 [file Image5.TIF]
